# Surgical Outcomes of Nonmelanoma Skin Cancer Managed with Systematic Preoperative Reflectance Confocal Microscopy-Guided Margin Assessment: A Retrospective Cohort Study Comparing Wide Local Excision and Mohs Micrographic Surgery

**DOI:** 10.3390/diagnostics16121916

**Published:** 2026-06-20

**Authors:** Federico Venturi, Elisabetta Mazzotti, Carlotta Baraldi, Biagio Scotti, Camilla Reggiani, Barbara Corti, Elisabetta Magnaterra, Daniela Tassone, Emi Dika

**Affiliations:** 1 Department of Medical and Surgical Sciences (DIMEC), Alma Mater Studiorum University of Bologna, 40138 Bologna, Italy; 2 Oncologic Dermatology Unit, IRCCS Azienda Ospedaliero-Universitaria di Bologna, 40138 Bologna, Italy; 3 Pathology Unit, IRCCS Azienda Ospedaliero Universitaria di Bologna, 40138 Bologna, Italy; 4 Plastic Surgery Unit, IRCCS Azienda Ospedaliera-Universitaria di Bologna, 40138 Bologna, Italy

**Keywords:** nonmelanoma skin cancer, reflectance confocal microscopy, Mohs micrographic surgery, wide local excision, surgical margins, local recurrence, margin control, preoperative mapping, basal cell carcinoma, squamous cell carcinoma

## Abstract

**Background:** Reflectance confocal microscopy (RCM) enables noninvasive, high-resolution visualization of skin tumors and may improve preoperative margin assessment in nonmelanoma skin cancer (NMSC). However, its impact on surgical outcomes in routine clinical practice remains incompletely defined. **Objective:** To evaluate surgical outcomes of NMSC managed with systematic preoperative RCM-guided margin assessment, comparing wide local excision (WLE) and Mohs micrographic surgery (MMS). **Methods:** We conducted a retrospective study of 71 consecutive NMSC treated at a tertiary dermatologic oncology center. All tumors underwent RCM evaluation for diagnosis and preoperative margin mapping. Outcomes included positive margins after WLE, local recurrence, recurrence-free survival, and the number of Mohs stages. Associations were analyzed using Fisher’s exact tests and Firth penalized logistic regression. **Results:** Among 47 tumors treated with WLE, positive margins occurred in 10.6%. Among 24 MMS cases, 70.8% were cleared in a single stage. Local recurrence occurred in 14.9% of WLE-treated tumors and in none of the MMS-treated tumors (*p* = 0.087). All recurrences occurred in tumors initially demonstrated positive margins after WLE, despite subsequent re-excision and histologic clearance. In multivariable Firth regression, MMS was associated with a lower risk of recurrence (OR 0.13; 95% CI, 0.008–2.10). **Conclusions:** In this RCM-guided cohort, low margin positivity after WLE and high single-stage clearance in MMS suggest improved surgical accuracy and efficiency. Recurrence was confined to margin-positive tumors, supporting a margin-driven model of tumor control and highlighting RCM as a potential preoperative margin-control strategy.

## 1. Introduction

Nonmelanoma skin cancer (NMSC), encompassing basal cell carcinoma (BCC) and squamous cell carcinoma (SCC), represents the most common human malignancy worldwide [1,2]. The rising burden of NMSC, driven by cumulative ultraviolet radiation exposure, population aging, and improved detection, poses a significant and growing challenge to healthcare systems worldwide [1,2,3]. Surgical excision remains the cornerstone of NMSC treatment [1,4]. Wide local excision (WLE) with predefined clinical margins is the most widely employed approach, while Mohs micrographic surgery (MMS), which provides intraoperative complete peripheral and deep en face margin assessment, is recommended for high-risk tumors and anatomically sensitive locations [5,6]. MMS achieves the highest cure rates among surgical modalities, with 5-year local recurrence rates of approximately 2.8–3.8% compared with 4–5% for standard excision [5,6]. A recent propensity score–weighted cohort study of high-stage cutaneous SCC demonstrated significantly lower local recurrence (9.6% vs. 19.8%), nodal metastasis, and disease-specific death with MMS compared with WLE [7]. However, MMS is resource-intensive, requires specialized training, and is not universally available, underscoring the need for strategies that can optimize surgical outcomes across treatment modalities.

A critical determinant of oncologic outcome in NMSC surgery is the achievement of clear surgical margins. Positive margins after initial excision are consistently associated with increased risk of local recurrence and may necessitate re-excision or adjuvant therapy [8,9]. Conventional preoperative margin assessment relies on clinical inspection and dermoscopy, yet these methods have recognized limitations [10,11,12,13]. Reflectance confocal microscopy (RCM) has emerged as a promising noninvasive imaging modality that provides near-histological, real-time visualization of skin microarchitecture at cellular resolution [14,15]. RCM enables in vivo identification of tumor nests, delineation of subclinical lateral extension, and differentiation of histologic subtypes, with diagnostic sensitivity and specificity exceeding 80% across multiple studies [16]. Preoperative RCM-guided margin mapping has demonstrated high correlation with histopathologic findings, with agreement rates of up to 91% in presurgical BCC evaluation [17,18]. A multicenter randomized controlled trial showed that an RCM-based one-stop-shop approach achieved 100% tumor-free margins after BCC excision, establishing noninferiority to standard care [19]. Furthermore, the integration of dermoscopy and RCM significantly enhances preoperative BCC subtype classification, with combined sensitivity reaching 81.4% and specificity 98.3% for superficial BCC [20]. Despite this growing evidence, the clinical impact of systematic preoperative RCM-guided margin assessment on surgical outcomes, including margin positivity rates, local recurrence, and the number of Mohs stages, remains insufficiently characterized in routine clinical practice. The aim of this study was to evaluate the surgical outcomes of NMSC managed with systematic preoperative RCM-guided margin assessment, comparing WLE and MMS in a consecutive cohort of patients treated at a single academic center. Specifically, we sought to assess the rates of positive surgical margins, local recurrence, and recurrence-free survival, and to explore the association between margin status and oncologic outcomes in the context of RCM-integrated surgical planning. It should be noted that, in the absence of a non-RCM control group, the present study is designed to evaluate clinical surgical outcomes, including margin positivity, number of Mohs stages, and local recurrence, in the context of systematic RCM use, rather than to establish a causal relationship between RCM-guided margin assessment and improved outcomes. Accordingly, the findings should be interpreted as supportive but not definitive evidence of benefit.

## 2. Materials and Methods

**Figure 1:**
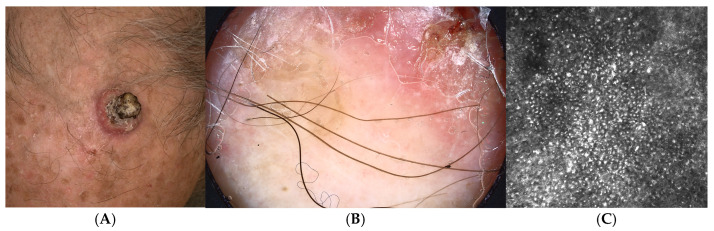
Reflectance confocal microscopy–guided presurgical margin assessment in cutaneous squamous cell carcinoma: Clinical image of a cutaneous squamous cell carcinoma located on the scalp in a 68-year-old male patient candidate for Mohs micrographic surgery (**A**). Dermoscopic examination of the peripheral tumor margin did not reveal evident dermoscopic criteria of squamous cell carcinoma (**B**). However, reflectance confocal microscopy demonstrated extensive confocal features consistent with subclinical SCC extension, leading to enlargement of the planned surgical margin (**C**). RCM-guided margin expansion allowed complete tumor clearance in a single Mohs stage. Scale bar = 100 µm.

We conducted a retrospective observational study including consecutive patients with histologically confirmed NMSC managed at the Oncologic Dermatology Unit, IRCCS Azienda Ospedaliero-Universitaria of Bologna, Policlinico of Sant’Orsola. All cases diagnosed between January 2020 and October 2023 were screened for inclusion. Eligible tumors were required to have undergone evaluation with RCM at diagnosis and before surgical treatment. Tumors lacking RCM assessment or follow-up information were excluded. The study was conducted in accordance with the Declaration of Helsinki and approved by the institutional review board. RCM was systematically employed as part of routine clinical practice for both diagnostic confirmation and preoperative tumor mapping. RCM examination was performed prior to surgery to confirm tumor diagnosis, delineate subclinical lateral tumor extension, assist surgical planning and margin definition. Lesion borders identified by RCM were marked clinically and used to guide surgical excision [21]. RCM findings were systematically integrated into surgical decision-making, and all tumors underwent preoperative margin assessment using RCM, thereby functioning as a noninvasive margin-control strategy across the entire cohort. Surgical approach was determined during multidisciplinary clinical evaluation, integrating clinical examination, dermoscopy, and RCM findings (tumors demonstrating poorly defined or irregular confocal borders, subclinical extension beyond the clinically estimated margin, or confocal features suggestive of aggressive histologic subtypes were preferentially allocated to MMS). Two surgical strategies were employed. WLE: Conventional excision was performed using margins defined according to clinical, dermoscopic, and RCM mapping. Surgical specimens underwent standard histopathologic evaluation, and margin status was classified as positive or negative. MMS: Mohs surgery was performed in tumors considered high-risk based on anatomic location, clinical features, histologic subtype, or RCM evidence of poorly defined tumor borders. MMS was conducted using staged excision with intraoperative microscopic margin control until tumor clearance was achieved (Figure 1).

The following variables were collected: age at diagnosis, sex, tumor size, anatomic site (head and neck vs. other sites; head and neck subsites recorded separately), primary vs. clinically recurrent tumor, histologic diagnosis (basal cell carcinoma or squamous cell carcinoma), and histologic subtype. Histologic variants were categorized as aggressive when infiltrative, morphoeic, basosquamous, micronodular, or other high-risk subtypes were present [1,22]. Adjuvant and neoadjuvant systemic therapies were recorded when administered.

The primary outcome was local recurrence during follow-up. Secondary outcomes included: positive surgical margins after initial WLE, recurrence-free survival, number of Mohs surgical stages required for tumor clearance.

Follow-up time was calculated from histologic diagnosis to the last clinical evaluation. Local recurrence was defined as histologically confirmed tumor reappearance at the site of a previously treated lesion during follow-up. Follow-up information was obtained from institutional medical records and routine dermatologic surveillance visits.

### 2.1. RCM Margin-Mapping Protocol

Preoperative RCM margin mapping was performed using the VivaScope 1500 console-based device (Caliber I.D., Inc., Rochester, NY, USA; MAVIG GmbH, Munich, Germany), which provides a lateral resolution of approximately 0.5–1 µm, axial resolution of 3–5 µm, and imaging depth of approximately 200–250 µm [23,24]. For lesions located on anatomically challenging sites (periocular region, nasal ala, ear) where the console-based device could not achieve adequate probe contact, the handheld VivaScope 3000 (Caliber I.D., Inc., Rochester, NY, USA; MAVIG GmbH, Munich, Germany) was used, which offers equivalent resolution with superior ergonomic adaptability for curved or concave surfaces [25,26]. Image acquisition followed a standardized protocol aligned with international expert recommendations [27]. For each lesion, baseline mosaics (0.5 × 0.5 mm individual optical sections stitched into up to 8 × 8 mm composite images) were acquired at five standard depth levels: stratum corneum, mid-epidermis, lower epidermis/dermoepidermal junction (DEJ), papillary dermis, and upper reticular dermis. For BCC, two additional mosaics were acquired at the corneal layer and reticular dermis to maximize the detection of basaloid tumor islands. For SCC, at least two dermal mosaics were obtained, with one including the reticular dermis when image quality permitted. The clinically and dermoscopically estimated tumor border was first marked on the skin with adhesive surgical tapes [21]. A peripheral scanning position was considered ‘confocal-positive’ (indicating subclinical tumor extension) based on the following criteria: for BCC, the presence of basaloid tumor islands, nests, or silhouettes, cord-like structures, or streaming/palisading patterns at the DEJ or papillary dermis; for SCC, the presence of atypical keratinocyte disarray, architectural disruption of the honeycomb pattern, or keratin pearls at the epidermal or dermal level [28,29]. These features have been validated in prior studies demonstrating 91–92% correlation between RCM-defined margins and histopathologic findings [29,30]. When confocal-positive features were identified beyond the initial clinical margin, the surgical marking was expanded radially until two consecutive confocal-negative scanning positions were achieved. The final surgical incision line was placed beyond the last identifiable confocal tumor feature.

### 2.2. Statistical Analysis

Continuous variables were summarized as median and interquartile range (IQR) and compared using the Mann–Whitney U test. Categorical variables were reported as counts and percentages and compared using Fisher’s exact tests. Because no recurrences occurred in the MMS group, resulting in statistical separation, associations between surgical approach and recurrence were evaluated using Firth penalized logistic regression, providing odds ratios (ORs) and 95% confidence intervals (CIs).

Predictors of positive margins after WLE were analyzed using univariable Firth logistic regression models.

The association between surgical margin status and local recurrence was specifically evaluated to explore a margin-driven model of tumor control. All tests were two-sided, and *p*-values < 0.05 were considered statistically significant. Statistical analyses were performed using Stata/SE version 18.0 (StataCorp LLC, College Station, TX, USA).

## 3. Results

71 patients with NMSC were included, comprising 47 tumors treated with WLE and 24 treated with MMS. Baseline clinicopathologic characteristics are summarized in Table 1.

Median age at diagnosis was 71.0 years (IQR, 59.0–76.5) in the WLE group and 69.5 years (IQR, 56.0–74.2) in the MMS group (*p* = 0.519). Most tumors were located on the head and neck (83.0% WLE vs. 75.0% MMS; *p* = 0.531), with the nose and periocular region representing the most frequent subsites. BCC represented the majority of tumors (80.9% WLE vs. 83.3% MMS; *p* = 1.000). Aggressive histologic subtypes were not underrepresented in the MMS group (14.9% WLE vs. 25.0% MMS; *p* = 0.340), indicating comparable baseline oncologic risk between surgical approaches. Median tumor size was similar between groups (1.5 cm [IQR, 0.6–2.0] vs. 2.0 cm [IQR, 0.6–2.0]; *p* = 0.603).

Among tumors treated with WLE, positive surgical margins after first-line surgery were observed in five of 47 cases (10.6%). Overall, the rate of margin positivity after initial excision was low. This finding likely reflects the systematic use of RCM for preoperative lesion delineation and margin assessment.

Among tumors treated with MMS, the median number of stages required for tumor clearance was 1. Notably, 70.8% of tumors were completely excised in a single stage.

During follow-up, local recurrence occurred in seven patients treated with WLE (14.9%) and in none of the patients treated with MMS (0%) (Fisher’s exact test, *p* = 0.087). No clinically meaningful difference in follow-up duration was observed between treatment groups. Among the 47 tumors treated with WLE, positive histologic margins after the initial excision were observed in five cases (10.6%). All five tumors subsequently underwent additional surgery and achieved histologic clearance. Nevertheless, all five later developed local recurrence during follow-up. Two additional recurrences occurred among tumors with negative histologic margins after the initial WLE procedure.

Median follow-up duration was 15 months (IQR, 11–19) in the WLE group and 18 months (IQR, 15.5–22.8) in the MMS group. Therefore, the absence of recurrences in the MMS cohort was not attributable to shorter surveillance time.

A strong association between surgical margin status and local recurrence was observed. Tumors with positive margins after the first excision demonstrated a markedly higher risk of recurrence during follow-up despite subsequent complete re-excision.

To account for separation resulting from the absence of recurrences in the MMS group, Firth penalized logistic regression was performed. Given the limited number of events, regression analyses were considered exploratory and hypothesis-generating. After adjustment for tumor size, aggressive histologic subtype, and clinically recurrent lesions, MMS remained associated with a lower risk of local recurrence (OR 0.13; 95% CI, 0.008–2.10; Table 2).

Exploratory Firth logistic regression analyses identified trends toward increased risk of positive margins with larger tumor size (OR 1.52 per cm; 95% CI, 0.81–2.85), and clinical ulceration, although no predictors reached statistical significance.

Stratification by tumor type revealed that BCC accounted for 58 of 71 tumors, with five cases (8.6%) showing positive margins after initial excision and seven cases (12.1%) developing local recurrence. By contrast, no positive margins or local recurrences were observed among the 13 SCCs included in the study. When stratified by histologic subtype, aggressive histotypes (n = 13) showed one positive margin (7.7%) and one local recurrence (7.7%), whereas non-aggressive histotypes (n = 58) showed four positive margins (6.9%) and six local recurrences (10.3%). These findings should be interpreted cautiously, given the limited number of SCC cases and the relatively short follow-up available for some patients.

## 4. Discussion

The present study evaluated the surgical outcomes of NMSC managed with systematic preoperative RCM-guided margin assessment, comparing WLE and MMS in a consecutive cohort treated at a single academic center. MMS was preferentially selected for tumors considered higher-risk based on anatomic location, histologic subtype, RCM findings, or recurrence history. This non-randomized allocation introduces confounding by indication. Notably, this selection bias would be expected to bias against MMS in the recurrence analysis, as higher-risk tumors were preferentially treated with MMS. The absence of recurrences in the MMS group despite the inclusion of clinically higher-risk tumors represents an interesting observation. However, given the retrospective design, non-randomized treatment allocation, and the possibility of residual confounding, this finding should be interpreted as an observed association within this cohort rather than evidence of treatment superiority.

The principal findings include a low rate of positive surgical margins after WLE (10.6%), a high proportion of single-stage Mohs clearance (70.8%), no local recurrences in the MMS group, and a strong association between margin positivity and local recurrence.

The incomplete excision rate observed in the WLE group compares favorably with previously reported data, particularly considering the predominance of head and neck tumors and the inclusion of aggressive histologic subtypes [31,32,33,34,35]. This finding likely reflects the systematic integration of RCM into preoperative surgical planning. By enabling in vivo visualization of subclinical tumor extension, RCM allows more accurate lateral margin delineation prior to excision. These results are consistent with a growing body of evidence supporting the use of RCM for presurgical mapping. Previous studies have demonstrated high concordance between RCM-defined margins and histopathologic findings, as well as improved diagnostic confidence and margin assessment in keratinocyte carcinomas. Despite known limitations, including limited imaging depth, operator dependency, and reduced performance in nonpigmented or deeply invasive lesions, RCM appears to provide clinically meaningful information for surgical planning. Taken together, these findings support the concept of RCM as a preoperative margin-control strategy rather than a purely diagnostic imaging tool [17,19,36,37].

### 4.1. Mohs Surgical Stages and Efficiency

The finding that 70.8% of tumors treated with MMS were cleared in a single stage is noteworthy. Although direct comparisons are limited by the absence of a non-RCM control group, this result is consistent with the hypothesis that preoperative RCM mapping may improve surgical efficiency by enabling more accurate initial margin delineation. Similar benefits have been reported in other settings, where imaging-guided presurgical mapping reduced the number of surgical stages compared with conventional clinical assessment [36,38]. In contrast, dermoscopy alone has not consistently demonstrated such an effect, suggesting that cellular-level imaging may provide additional value in defining tumor boundaries [39].

### 4.2. Local Recurrence and the Margin-Driven Model of Tumor Control

Local recurrence occurred in 14.9% of WLE-treated tumors and in none of the MMS-treated tumors. Although this difference did not reach statistical significance, the trend is consistent with the established superiority of MMS for margin control.

A key observation in the present study is that all tumors initially demonstrating positive histologic margins after WLE subsequently developed local recurrence, despite subsequent re-excision and histologic clearance. However, recurrence was not exclusively confined to this subgroup, as two additional recurrences occurred among tumors that achieved negative margins after the initial excision. This finding may identify a subset of biologically or surgically challenging lesions characterized by greater subclinical extension, infiltrative growth, or more complex three-dimensional spread patterns.

Although RCM improves lateral margin delineation, its limited penetration depth may reduce accuracy in tumors with deeper infiltrative components. Consequently, tumors requiring re-excision after initial positive margins may represent lesions in which subclinical extension exceeded the spatial resolution achievable with preoperative confocal assessment. This is consistent with the well-established association between positive margins and recurrence risk [40,41]. Standard histopathologic evaluation after conventional excision samples only a limited portion of the surgical margin, which may lead to underdetection of residual tumor. In this context, strategies that improve preoperative margin delineation, such as RCM, may have a direct impact on clinical outcomes. In fact, all re-excisions in the WLE group were evaluated using standard bread-loaf histopathologic sectioning, which examines only a small fraction (approximately 1–2%) of the surgical margin [42,43]. This sampling limitation means that ‘clear’ re-excision margins on bread-loaf histology do not guarantee complete tumor removal, as residual tumor in unsampled margin areas may have been missed. Indeed, a longitudinal study of 161 patients with positive margins after initial NMSC excision found that 48% had residual tumor upon re-excision, while among those not re-excised, 6.6% developed recurrence at 5 years [44]. This observation further supports the rationale for comprehensive margin assessment techniques such as MMS, which visualize the entire marginal surface, especially when combined with non-invasive diagnostic techniques. The precise anatomic relationship between the site of recurrence and the prior positive margin edge was not systematically recorded, which represents a limitation of this retrospective analysis.

The Firth penalized logistic regression analysis, performed to account for complete separation due to the absence of recurrences in the MMS group, showed a consistent protective effect of MMS after adjustment for tumor size, histologic subtype, and clinically recurrent lesions. Although confidence intervals were wide, the direction and magnitude of the association support the observed clinical signal.

The observation that all positive margins and all recurrences occurred exclusively among BCCs, with no events among the 13 SCCs, is noteworthy but must be interpreted with caution, given the small SCC sample size. A large meta-analysis of over 128,000 keratinocyte carcinomas reported pooled incomplete excision rates of 11.0% (95% CI, 9.7–12.4%) for BCC and 9.4% (95% CI, 7.6–11.4%) for SCC without imaging guidance [45]. The 8.6% incomplete excision rate for BCC in the present RCM-guided cohort compares favorably with this benchmark. The absence of positive margins among SCCs may reflect the smaller sample size, the generally better-defined clinical borders of SCC compared to BCC, or the effectiveness of RCM in delineating SCC margins in this cohort, although RCM sensitivity for SCC-specific features has been reported as lower than for BCC (67% vs. 92%) [46]. Additionally, BCC was significantly more likely to exhibit positive resection margins during MMS compared to SCC in a study of 151 tumors, with high-risk BCC subtypes showing the highest probability of margin involvement [47]. However, the limited number of recurrence events resulted in wide confidence intervals around regression estimates. Consequently, multivariable analyses should be considered exploratory and hypothesis-generating rather than definitive estimates of treatment effect. Nevertheless, the direction and magnitude of the observed associations were consistent with the absolute event counts observed in the cohort.

### 4.3. Predictors of Positive Margins

Exploratory analyses identified trends toward increased risk of positive margins with larger tumor size and clinical ulceration, although no predictors reached statistical significance. These findings are consistent with the literature. The inability to identify statistically significant predictors in the present cohort likely reflects the small sample size and the overall low event rate, which may itself be attributable to the RCM-guided approach. Although the present study did not include molecular characterization, the observed tendency toward recurrence in a subset of surgically challenging tumors may reflect underlying biologic heterogeneity beyond purely morphologic features. Increasing evidence supports a role for non-coding RNA networks and microRNA-related pathways in modulating tumor aggressiveness and progression in cutaneous oncology [48,49,50,51].

### 4.4. Clinical Implications

Among noninvasive imaging modalities approved by the US FDA and EMA for skin cancer assessment, RCM provides the highest lateral resolution (~0.5–1 µm) with axial resolution of 3–5 µm, enabling near-histological cellular-level visualization [24,52,53]. However, its practical imaging depth is limited to approximately 200–250 µm, with significant image quality degradation beyond this threshold. This depth limitation is clinically relevant, as deeper infiltrative BCC components and invasive SCC extending beyond the papillary dermis may escape detection. By contrast, optical coherence tomography (OCT) provides lower lateral resolution (~7.5 µm for conventional OCT) but images to a depth of approximately 1–2 mm, offering complementary depth information [54]. Agreement between OCT-defined margins and histopathology ranges from 84% to 95.5%, and a systematic review of 11 studies (303 patients) demonstrated that OCT can reduce MMS stages (from 1.89 to 1.23) and enable narrower excision margins [54,55]. High-frequency ultrasound (HFUS) at 20–50 MHz provides depth assessment up to several millimeters with a resolution of 50–200 µm, useful for tumor thickness measurement but lacking cellular resolution. Dermoscopy-guided HFUS (DG-HFUS) outperformed HFUS alone, achieving 82.4% sensitivity and 91.3% specificity for distinguishing aggressive from low-risk BCC subtypes [52]. Line-field confocal OCT (LC-OCT), combining confocal and OCT principles, achieved the highest accuracy for BCC subtyping in a recent systematic review, with 100% sensitivity for infiltrative BCC. These modalities are complementary rather than competing: RCM excels at superficial lateral margin delineation with cellular resolution, while OCT and HFUS provide superior depth assessment. Importantly, RCM holds approval by both the US FDA and the EMA, facilitating its integration into routine preoperative assessment, which represents a practical strategy to optimize surgical outcomes across both WLE and MMS. For WLE, RCM-guided margin mapping may reduce the rate of incomplete excisions, thereby decreasing the need for re-excision and potentially lowering recurrence risk. For MMS, preoperative RCM delineation may improve procedural efficiency by reducing the number of stages required for tumor clearance, with implications for operative time, cost, and tissue conservation. These benefits are particularly relevant for tumors in cosmetically and functionally sensitive areas, where tissue preservation is paramount. In settings where MMS is not available, RCM-guided WLE may serve as a valuable alternative strategy to enhance margin control, although this hypothesis requires validation in larger, prospective, controlled studies.

The present findings should be contextualized alongside other imaging-guided margin assessment strategies. Alawi et al. demonstrated that OCT-defined lateral margins correctly indicated complete tumor removal in 84% of NMSC cases (n = 19 lesions), and surgical margins chosen by the surgeon never fell below the OCT-defined margin [55]. A 2026 systematic review of 11 studies (303 patients) reported OCT–histopathology agreement of 84–95.5% for margin assessment, with demonstrated reductions in MMS stages [54]. Boostani et al. reported that dermoscopy-guided HFUS outperformed standard HFUS for BCC margin and subtype assessment, achieving 82.4% sensitivity and 91.3% specificity for identifying aggressive subtypes [56,57]. While direct comparison across studies is limited by heterogeneous protocols and populations, the 10.6% margin positivity rate observed in the present RCM-guided WLE cohort compares favorably with published incomplete excision rates of 5–17% for standard WLE without imaging guidance. The complementary strengths of these modalities suggest that multimodal imaging strategies may further optimize presurgical planning in NMSC.

Nevertheless, implementation of RCM-guided surgical planning requires dedicated equipment, operator expertise, and integration into clinical workflows, factors that may limit widespread adoption outside specialized referral centers. Future studies evaluating cost-effectiveness and implementation strategies are warranted.

### 4.5. Limitations

This study has several limitations. Its retrospective, single-center design may limit generalizability and introduce potential selection bias. Treatment allocation was not randomized but based on clinical decision-making integrating RCM findings. The sample size was limited, resulting in low statistical power and wide confidence intervals. In addition, the absence of a non-RCM control group precludes definitive attribution of outcomes to RCM use. Detailed operative metrics, including procedure duration, defect dimensions, and reconstruction type, were not systematically recorded in this retrospective cohort and represent a limitation of this analysis. Future prospective studies should capture these variables to fully characterize the translational impact of RCM-guided surgical planning on operative efficiency and resource utilization.

## 5. Conclusions

In this consecutive cohort of NMSC managed with systematic preoperative RCM-guided margin assessment, the rate of positive surgical margins after WLE was low compared with published benchmarks, the majority of MMS cases were cleared in a single stage, and no recurrences occurred in the MMS group. Tumors that initially demonstrated positive histologic margins after WLE showed an increased risk of subsequent recurrence despite re-excision and histologic clearance, suggesting the presence of biologically or surgically challenging lesions characterized by greater subclinical extension. These findings suggest that the systematic integration of RCM into preoperative surgical planning is associated with favorable rates of margin positivity and surgical efficiency in NMSC management. Prospective, controlled, multicenter studies with longer follow-up are warranted to confirm these observations and to define the optimal role of RCM in the surgical workflow for keratinocyte carcinomas.

## Figures and Tables

**Table 1:** Baseline characteristics according to surgical approach.

Variable	WLE (*n* = 47)	MMS (*n* = 24)	*p* Value
Age, years median (IQR)	71.0 (59.0–76.5)	69.5 (56.0–74.2)	0.519
Male sex, *n* (%)	26 (55.3)	6 (25.0)	0.023
Tumor size, cm median (IQR)	1.5 (0.6–2.0)	2.0 (0.6–2.0)	0.603
Head & neck location, *n* (%)	39 (83.0)	18 (75.0)	0.531
Nose	13	10	
Face (other)	14	5	
Periocular	4	2	
Scalp	4	0	
Ear	3	1	
Neck	1	0	
Basal cell carcinoma, *n* (%)	38 (80.9)	20 (83.3)	1.000
Aggressive histotype, *n* (%)	7 (14.9)	6 (25.0)	0.340
Follow-up, months median (IQR)	15 (11–19)	18 (15.5–22.8)	

**Table 2:** Firth penalized logistic regression for local recurrence.

Variable	OR	95% CI
MMS vs. WLE	0.13	0.008–2.10
Tumor size (per cm)	1.52	0.81–2.85
Aggressive histotype	0.29	0.02–4.35
Clinically recurrent lesion	3.28	0.58–18.61

## Data Availability

The data that support the findings of this study are available on request from the corresponding author.

## Abbreviations

The following abbreviations are used in this manuscript:

NMSC	Nonmelanoma skin cancer
BCC	Basal cell carcinoma
SCC	Squamous cell carcinoma
WLE	Wide local excision
MMS	Mohs micrographic surgery
RCM	Reflectance confocal microscopy
ORs	Odds ratios
OCT	optical coherence tomography
HFUS	High-frequency ultrasound
